# Treatment and prognosis of Scimitar syndrome: A retrospective analysis in a single center of East China

**DOI:** 10.3389/fcvm.2022.973796

**Published:** 2022-08-24

**Authors:** Kai Wang, Xinyi Xu, Tingliang Liu, Wei Gao, Ying Guo

**Affiliations:** ^1^Department of Cardiology, Heart Center, Shanghai Children's Medical Center, School of Medicine, Shanghai Jiao Tong University, Shanghai, China; ^2^Department of Pediatrics, The First Affiliated Hospital of Wenzhou Medical University, Wenzhou Medical University, Wenzhou, China

**Keywords:** Scimitar syndrome, congenital heart disease, interventional therapy, surgery treatment, prognosis

## Abstract

**Background:**

Scimitar syndrome is a rare congenital cardiovascular malformation; its optimal management remains controversial. This study aims to present the clinical experience of this disease in our center.

**Methods:**

We undertook a retrospective review of 34 patients with Scimitar syndrome documented at our institution between January 2013 and December 2018. The patients' clinical characteristics, management, and prognosis data were collected and analyzed.

**Results:**

Thirty-four patients, including 16 males and 18 females, were enrolled with a median age at diagnosis of 7 months and a follow-up period of 22.5 months. The infantile form of Scimitar syndrome presents more tendency for pulmonary hypertension (PH), pulmonary vein stenosis (PVS), and mortality than the adult form. Of the 15 patients who underwent surgical correction of the Scimitar vein, four had post-operation PVS. There was no significant difference in the stenosis incidence between baffle repair and Scimitar vein reimplantation groups. Eight patients received interventional catheter therapy, including occlusion of aortopulmonary collateral arteries (APCs) and other intracardiac malformations, without the following surgery. The overall mortality rate was 20.5% (7 of 34) over the study period. High-risk factors of death included age at diagnosis (*p* = 0.000), PH (*p* = 0.007) and PVS (*p* = 0.014).

**Conclusions:**

Infantile Scimitar syndrome needs intense suspicion for early diagnosis and multidisciplinary treatment. Interventional treatment of Scimitar syndrome alleviates pulmonary artery pressure and progression during infancy. Baffle repair and direct reimplantation of the Scimitar vein used in the surgical treatment of Scimitar syndrome are safe and have similar effects. Age at diagnosis, PH, and PVS are high-risk factors for death in Scimitar syndrome.

## Introduction

**Figure 1:**
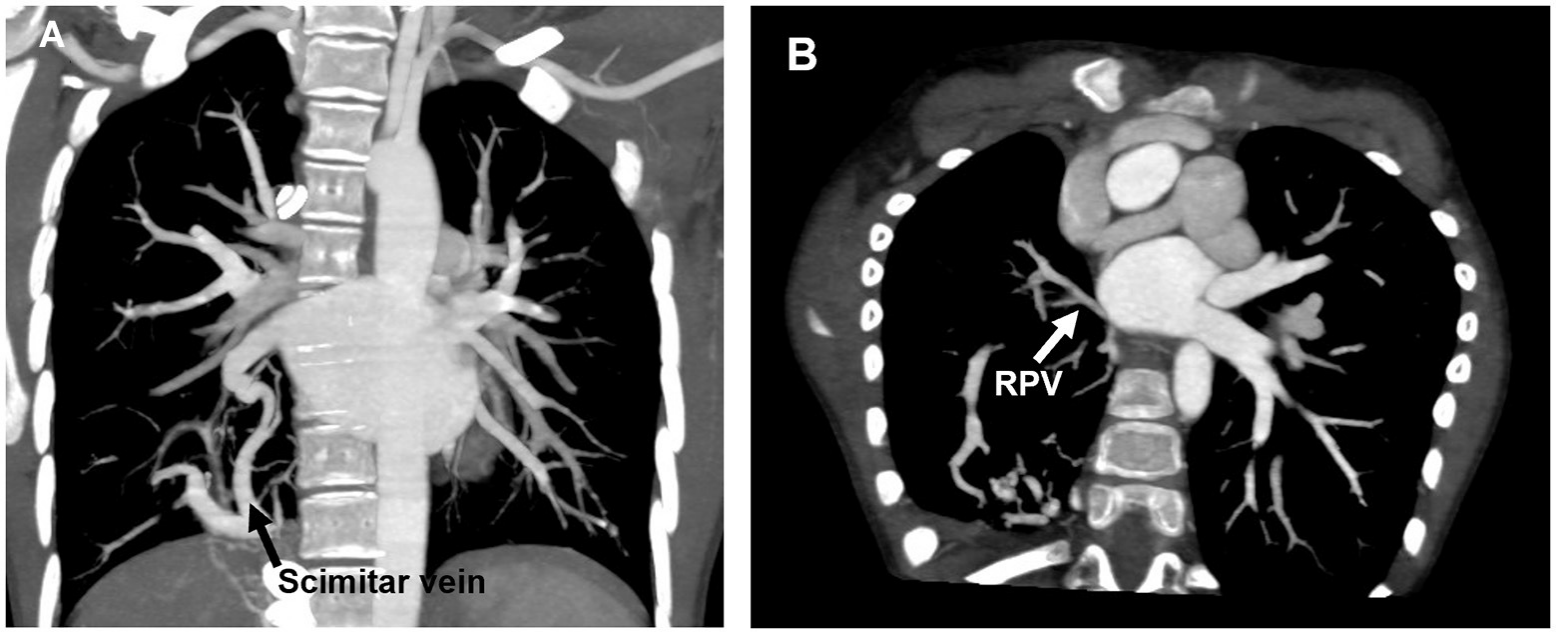
CT scan showing anomalous pulmonary venous drainage of the right lung to the IVC **(A)** and restenosis of RPV after operation **(B)**. CT, Computed tomography; IVC, Inferior vena cava; RPV, Right pulmonary vein.

Scimitar syndrome is a rare congenital cardiopulmonary anomaly with an incidence of approximately 1–3/100,000 live births (1). It is characterized by partial or entire unilateral anomalous pulmonary venous drainage into the inferior vena cava (IVC), which is often accompanied by abnormal arterial supply from descending aorta to the right hypoplastic lung, dextraposition of the heart (2, 3). Since the first report of Scimitar syndrome in 1836 (4, 5), the term “Scimitar” indicates the radiographic Turkish curvilinear appearance of the anomalous venous drainage, likened to the silhouette of an oriental sword (Figure 1A). Intracardiac defects and aortopulmonary collaterals are commonly associated with Scimitar syndrome, with probably a 40% prevalence (6).

Symptoms of Scimitar syndrome vary from different individuals, commonly including cyanosis, respiratory distress, tachypnea, recurrent pneumonia, and heart failure. According to the onset age of symptoms, Scimitar symptom has been divided into infantile form (during infancy) and adult form (1). The infantile form presents symptoms early and is more likely to require treatment, causing a picture of a severely ill and agitated patient with a mortality rate of up to 45% (3). In contrast, the adult form is usually asymptomatic with incidental findings and rarely needs emergent intervention (6). Patients with the infantile form often have severe clinical symptoms, including pulmonary hypertension, congestive heart failure, and a worse prognosis. Therefore, a multidisciplinary approach is urgently required. The treatment of Scimitar syndrome mainly includes a catheter-based obligation of the abnormal systemic arteries from the aorta to the right lung and surgical correction by either baffling a tunnel or a direct re-implantation to the left atrium (LA) of the anomalous pulmonary venous drainage.

To date, few publications have reported the clinical outcomes of Scimitar syndrome due to rare morbidity; most of these case reports focused on the infantile form of this syndrome with a limited population (7–10). However, the interventional period and therapeutic strategy of Scimitar syndrome still have not reached a consensus. Therefore, this study aimed to present the experience with Scimitar syndrome encountered in our center in East China and to evaluate the optimal treatment options and high-risk factors of post-operation complications and death in patients with Scimitar syndrome.

## Materials and methods

### Patients

From January 2013 to December 2018, 34 patients diagnosed with Scimitar syndrome in Shanghai Children's Medical Center (SCMC) were enrolled in this study. Inclusion criteria consisted of partial anomalous pulmonary venous connection to the IVC. There were 16 males and 18 females with a median age of 7 months (range from 3 days to 10 years old). The median follow-up period was 22.5 months. Clinical data, including symptoms, signs, electrocardiogram (ECG), chest X-ray, transthoracic echocardiography (TTE), cardiac multidetector computed tomography (MDCT), cardiac catheterization, surgical treatment, and outcomes were collected and analyzed retrospectively. All protocols and procedures performed in this study were approved by the Ethics Committee of the SCMC, and informed consent was obtained from each patient's guardians. All methods were performed in accordance with the relevant guidelines and regulations; all procedures were performed in accordance with the Declaration of Helsinki.

### Hemodynamics evaluation

The pulmonary-to-systemic blood flow ratio (Qp/Qs) and the pulmonary-to-systemic pressure ratio (Pp/Ps) were quantified by cardiac catheterization. The systolic pulmonary artery pressure was characterized as being below or above 50% of the systemic level (11). We defined significant pulmonary hypertension (PH) as Pp/Ps > 0.5, tricuspid regurgitation velocity>3.5 m/s, and ventricular septum position deviated to the left. Pulmonary venous stenosis (PVS) was defined by non-phasic flow or velocity >1.6 m/s from spectral Doppler in the pulmonary vein by echocardiography (11), or discrete stenosis of the pulmonary veins during angiography, or obvious morphology baffle or venous stenosis detected by cardiac MDCT. The presence and the blood supply of aortopulmonary collateral arteries (APCs) were assessed by catheterization or cardiac MDCT.

### Statistical analysis

Continuous variables were described as mean±SD or median (range). Categorical variables were described by counts and percentages. *x*^2^ test was used for the evaluation of binary variables. Univariate analysis was used for high-risk factors of death. Multivariate analysis was prohibited by insufficient population and mortality events. A *p-value* < 0.05 was considered statistical significance. Analyses were performed using SPSS 19.0 software.

## Results

### Demographic data and clinical presentation

The clinical characteristics of the patients are illustrated in Table 1. Totally 22 patients were diagnosed at the age within 1 year (infantile form), and 12 patients were diagnosed at an older age (adult form). There were 19 Scimitar syndrome patients complicated with extra-cardiac malformations, including four cases of congenital diaphragmatic hernia, one case of scoliosis, one case of pectus excavatum, seven cases of horseshoe lung, and six cases of tracheobronchial stenosis. Atrial septal defect (ASD) was detected in 24 patients, while 14 cases were associated with cardiac malformations other than ASD. The most common cardiac anomalies were patent ductus arteriosus (PDA, eight cases) and ventricular septal defect (VSD, four cases). APCs were confirmed in 27 patients (79.4%) by echocardiography, MDCT, or catheterization. Compared with the adult form, the infantile form patients had a higher risk of developing PH (*p* = 0.035), pulmonary vein stenosis (PVS, *p* = 0.006), and mortality rate (*p* = 0.036). Besides, both extra- and intra-cardiac malformations were more common in infantile form compared with those in adult form patients. Similar results can be found in comparing APCs between infantile and adult forms without a statistical difference (*p* = 0.211). Two patients associated with severe tracheal stenosis gave up following treatments by their parents, and another two with Eisenmenger syndrome only received anti-PH therapy without further interventional or surgical treatment.

**Table 1:** Demographic and clinical characteristics of the study population.

**Variable**	**Infantile *n* = 22**	**Adult *n* = 12**	***P* value**
**Gender**			
Male, *n* (%)	12 (75.0)	4 (25.0)	0.236
Female, *n* (%)	10 (55.6)	8 (44.4)	
**Age**			
Symptomatology	3 months (1 day to 11 months)	32 months (12–123)	
Diagnosis	4 months (1 day to 11 months)	36 months (12–126)	
Family history, *n* (%)	5 (22.7)	2 (16.7)	0.677
**Symptoms**, ***n*** **(%)**			
Tachypnea	14 (63.4)	3 (25.0)	0.031
Heart failure	6 (27.3)	2 (16.7)	0.486
Pneumonia	5 (22.7)	5 (41.7)	0.247
Cyanosis	3 (13.6)	2 (16.7)	0.812
Extracardiac anomalies, *n* (%)	12 (54.5)	7 (58.3)	0.832
Complicated ASD, *n* (%)	16 (72.7)	8 (66.7)	0.077
**Cardiac anomalies excluding ASD**, ***n*** **(%)**	11 (50.0)	3 (25.0)	0.275
PDA + VSD	2		
PDA	5	1	
VSD	1	1	
DORV + VSD	1		
TOF		1	
VSD + CoA	1		
VSD + RVOTO	1		
APCs, *n* (%)	19 (86.4)	8 (66.7)	0.211
PH, *n* (%)	14 (63.6)	3 (25.0)	0.035
PVS, *n* (%)	10 (45.5)	1 (8.3)	0.006
Mortality, *n* (%)	7 (31.8)	0 (0)	0.036

APCs, Aortopulmonary collateral arteries; PH, Pulmonary hypertension; PVS, Pulmonary vein stenosis; ASD, Atrial septal defect; PDA, Patent ductus arteriosus; VSD, Ventricular septal defect; DORV, Double outlet of right ventricle; TOF, Tetralogy of Fallot; CoA, Coarctation of aorta; RVOTO, Right ventricular outflow tract obstruction.

All the patients had varying degrees of right pulmonary hypoplasia. As shown in Table 2, 25 patients (73.5%) with superior and inferior pulmonary veins from the right hypoplastic lung converged and drained into the inferior vena cava (IVC). In two patients (5.9%), the right superior pulmonary vein (RSPV) and the right inferior pulmonary vein (RIPV) flowed into IVC and right atrium (RA), respectively. Four patients (11.7%) with RSPV or RIPV flowed into IVC separately. In two patients (5.9%), RSPV and some branches of RIPV flowed into the IVC. Especially, the right pulmonary vein (RPV) descended through the vertical vein, connecting with the portal vein, and drained into IVC *via* a hepatic vein in one patient (2.9%). Meanwhile, 9 (26.5%) and 2 (5.9%) patients had pre-operative right PVS and left PVS, respectively.

**Table 2:** Different lesions of anomalous pulmonary venous drainage.

	**Number**
RSPV and RIPV converge and drain to IVC, *n* (%)	25 (73.5)
RSPV to IVC and RIPV to RA, respectively, *n* (%)	2 (5.9)
RIPV to IVC, *n* (%)	3 (8.8)
RSPV to IVC, *n* (%)	1 (2.9)
RSPV and branches of RIPV to IVC, *n* (%)	2 (5.9)
RPV descending through vertical vein connects portal vein and returns to IVC *via* a hepatic vein, *n* (%)	1 (2.9)
Right PVS, *n* (%)	9 (26.5)
Left PVS, *n* (%)	2 (5.9)

RSPV, Right superior pulmonary vein; RIPV, Right inferior pulmonary vein; IVC, Inferior vena cava; RA, Right atrium; RPV, Right pulmonary vein; PVS, Pulmonary vein stenosis.

### Interventional catheterization

Of the 27 patients who had APCs to the ipsilateral lung, 15 received coil occlusion of the APCs. Two patients had PDA occlusion simultaneously; one patient underwent surgical ligation during Scimitar vein surgery. Eight patients did not require the intervention of small APCs without hemodynamic significance. Of note, after the interventional procedure, there were eight patients whose pulmonary artery pressure decreased from 55.32 ± 5.62 mmHg to 31.23 ± 3.45 mmHg, with Qp/Qs alleviated below 1.3. During follow-up, these patients' clinical symptoms, pulmonary pressure, and right ventricular volume were alleviated to stable without requiring further interventional or surgical treatment.

### Surgical treatment

Seventeen patients underwent surgical repair for Scimitar syndrome, and the concomitant cardiac malformations were corrected simultaneously. An intra-atrial baffle or Scimitar vein reimplantation was used as surgical approach, mainly depending on anatomic and pathological features, and surgeon preference. With respect to IVC drainage in patients underwent intra-atrial baffle technique, a pericardium patch may be adopted to avoid IVC return obstruction if necessary.

There were seven and eight patients received an intra-atrial baffle and Scimitar vein reimplantation, respectively. Comparisons between these two procedures were illustrated in Table 3. Except for median body weight at surgery, there was no statistically significant difference in age at surgery, cardiopulmonary bypass time, ICU staying time, and postoperative complications between these two surgical techniques. We found that three patients underwent post-operation PVS in the baffle group, while two of them already had PVS before the surgical management. Similarly, only one patient receiving reimplantation was found to have PVS, which existed pre-operation (Figure 1B). Meanwhile, we found that post-operative PVS was closely related to pre-operative PVS (*p* = 0.009).

**Table 3:** Patients' outcomes according to different surgical techniques.

**Variable**	**Intra-atrial baffle**	**Re implantation**	***P-value***
Median age at surgery (range), month	9 (1.5–120)	13.5 (2–84)	0.849
Males, *n* (%)	5 (71.4)	4 (50.0)	0.399
Median body weight at surgery (IQR), kg	12 (6–25)	7 (5–14)	0.048
Median CPB time (IQR), min	126 (106–145)	125 (103–158)	0.956
Median ICU stay (IQR), d	4 (2–7)	5 (2–8)	0.892
Hospital mortality, *n* (%)	1 (14.3)	0	0.268
PVS, *n* (%)	3 (42.9)	1 (12.5)	0.569

CPB, Cardiopulmonary bypass; IQR, Interquartile range; ICU, Intensive care unit; PVS, Pulmonary vein stenosis.

Two patients died of congestive heart failure several hours after the operation. One of them was a 2-month Scimitar syndrome infant accompanied with VSD and coarctation of aorta (CoA). It was found that RSPV of this patient was absent, combined with a tiny thin RIPV, which couldn't be corrected during the operation. Therefore, only CoA and VSD of this patient were repaired instead. The other was a 4-month-old patient, whose Scimitar vein connected to IVC with an obtuse angle and located posterior to hepatic vein. The distance between Scimitar vein and LA was far away, it was impossible to anastomose the Scimitar vein to the LA directly. On the other hand, a crest created inside of the conduit by baffle technique led to obstruction of IVC return. Since intra-atrial baffle and reimplantation failed to perform, an existing ASD (2 mm) augmentation was used as a palliative strategy instead.

### Scimitar vein obstruction

Pre-operative Scimitar vein obstruction was documented in 10 patients (29.4%). Among them, one patient with Eisenmenger syndrome didn't meet the indication for surgery at the time of diagnosis. Three patients died pre-operation. Two patients with pre-operative Scimitar vein obstruction whose pulmonary artery pressure decreased to normal after APCs occlusion did not need further surgery. These patients didn't present obvious clinical symptoms during follow-up by far. In terms of PVS, fibrous crest was resected from orifice of the Scimitar vein if existing. Besides, oblique incision of Scimitar vein was used to anastomose to LA as an option for augmentation of pulmonary vein. Four patients with Scimitar vein obstruction underwent surgical repair, among which one died of heart failure within 24 h after the operation. During follow-up, echocardiographs from three survived patients showed that pulmonary vein velocity mildly increased (2.0–2.58 m/s) but without clinical symptoms, so reoperation was unnecessary.

### Pulmonary hypertension (PH)

Since Scimitar syndrome patients may develop pulmonary hypertension during progression, we analyzed the initial hemodynamics, APCs, PVS, and outcomes of Scimitar syndrome patients accompanied with PH, as listed in Table 4. It showed that infantile form of Scimitar patients presented higher mean pulmonary artery pressure (MPAP) and more tendency of APCs, while there was no significant difference in PVS and mortality between these two forms. Advanced anti-PH therapy in the form of Bosentan with or without Sildenafil was started in 8 patients, among whom only one patient developed PVS and died 2 months after treatment.

**Table 4:** Clinical characteristics of PH patients with different forms.

**Variable**	**Infantile *n* = 14**	**Adult *n* = 3**	***P-*value**
MPAP (mmHg)	67 ± 7.8	35.4 ± 5.6	0.038
APCs, *n* (%)	14 (100)	2 (66.7)	0.026
PVS, *n* (%)	8 (57.1)	1 (33.3)	0.253
Mortality, *n* (%)	4 (28.6)	0	0.190

PH, Pulmonary hypertension; MPAP, Mean pulmonary artery pressure; APCs, Aortopulmonary collateral arteries; PVS, Pulmonary vein stenosis.

### Mortality and risk factors

Of the 34 patients diagnosed with Scimitar syndrome, 7 (20.6%) died. All the seven dead patients were infants under 4 months old. Three deaths of heart failure and one of tracheobronchial stenosis complicated with pulmonary infection; all died before the operations. The other three patients died post-operation, including two deaths within 24 h after the operation, and one case died due to left PVS-induced heart failure 2 months after the operation. Furthermore, univariate analysis revealed that age at diagnosis (*p* = 0.000), PH (*p* = 0.007), and PVS (*p* = 0.014) as risk associates (Table 5).

**Table 5:** Univariate analyses of risk factors for death.

**Variable**	**Death (*n* = 7)**	**Survival (*n* = 27)**	***P-value***
Male, *n* (%)	2 (28.6)	14 (51.9)	0.252
Age (month)	4.5 ± 3.2	51.7 ± 41.4	0.000
PH, *n* (%)	7 (100)	10 (37.0)	0.007
Extracardiac malformation, n (%)	1 (14.3)	13 (48.1)	0.198
PVS, *n* (%)	5 (71.4)	5 (18.5)	0.014
Cardiac anomalies excluding ASD, *n* (%)	4 (57.1)	10 (37.0)	0.410
APCs, *n* (%)	6 (85.7)	21 (77.8)	1.000

PH, Pulmonary hypertension; PVS, Pulmonary vein stenosis; APCs, Aortopulmonary collateral arteries.

## Discussion

Scimitar syndrome is a rare and complex congenital cardiopulmonary vascular disease with an estimated prevalence of 1–3 per 100,000 births (9). To our knowledge, this study is one of the retrospective analyses of Scimitar syndrome with the sizable population in a single center. In the present series, 34 Scimitar syndrome patients were enrolled with the median age at diagnosis of 7 months. Of 17 patients (50%) with PH, 8 and 15 received catheterization and surgical therapy, respectively. The overall mortality rate was 20.5%, and the mortality for infantile form was 31.8%, which is consistent with previous reports of infantile form of Scimitar syndrome (9, 12). We also found that the infantile form of Scimitar syndrome had more tendency of PH, PVS, and mortality than the adult form. What's more, high-risk factors for death included age at diagnosis, PH, and PVS.

The severity and clinical presentation of this condition are frequently determined by the age at which it is diagnosed, as well as the existence of other potentially complex congenital cardiac disorders. Patients with Scimitar syndrome are commonly complicated with a diaphragmatic hernia, horseshoe lung, or tracheobronchial stenosis. The clinical manifestations of Scimitar syndrome vary in severity (10, 11, 13–15); our data demonstrate that infantile Scimitar syndrome patients have a higher incidence of PH, PVS, and worse prognosis, especially those within 4 months old. Multiple factors include increased pulmonary blood flow, decreased pulmonary vascular bed due to unilateral lung dysplasia, congenital structural pulmonary abnormalities, and Scimitar vein stenosis account for PH. Furthermore, increased pulmonary blood flow can be contributed by anomalous pulmonary venous drainage, large left-to-right shunt lesions of congenital heart disease, and APCs.

Catheterization provides an accurate evaluation of anomalous pulmonary venous drainage, pulmonary arterial pressure, volume burden of pulmonary circulation, obstruction of pulmonary veins, hemodynamics of APCs, and associated structural cardiac anomalies. Moreover, catheterization is an interventional procedure for coil embolization for APCs and coexisting congenital heart disease, balloon, or stenting platy for vessel stenosis. Since large APCs may lead to congestive heart failure and recurrent pulmonary infection at an early stage, occlusion of APCs effectively reduces pulmonary artery pressure, improving symptoms or cardiac function. For those Scimitar syndrome patients associated with other cardiac malformations, preoperatively interventional embolization of APCs can effectively prevent the occurrence of “perfusion lung” (16, 17). In the present study, 27 (79.4%) patients presented with APCs, while 15 received APCs occlusion. Strikingly, we found that eight patients' pulmonary artery pressure decreased to normal (MPAP <25 mmHg) after interventional occlusion without symptoms, indicating interventional therapy candidate as an effective strategy for Scimitar syndrome treatment, even with no need for further surgery. These patients had no additional intracardiac anomalies and coil embolization of APCs were essential for Scimitar syndrome. However, further close follow-up is still needed for long term evaluation. Treatment and monitoring of Scimitar syndrome depend on the underlying pathology and the patient's symptoms. Recently reported studies also demonstrated that asymptomatic Scimitar syndrome patients who were detected in accident examination had a good prognosis even didn't receive any operation (3, 9, 10, 18).

The surgical approach to correct Scimitar syndrome varies with each case and surgeon preference, depending on the anatomic and pathologic features. However, the surgical indications of the Scimitar syndrome are still nebulous currently, especially for asymptomatic patients. Based on previous and present data, when a symptomatic patient is accompanied by PH and/or increased pulmonary blood flow (Qp/Qs > 1.5), PVS, surgical treatment may be a candidate as an effective treatment strategy (12, 19–21). Given that Scimitar syndrome can cause long-term physiologic repercussions if left untreated, surgical surgery for symptomatic patients may be a safe and effective therapy option (9). It's worth noting that the prognosis for Scimitar syndrome presenting within infancy is generally poor with or without surgery. It's reported that the 1-year survival for a patient diagnosed during infancy with symptomatic Scimitar syndrome is 62.5% if surgical correction is expeditiously performed. It may decrease to 54.5% if conservative management is undertaken (9). Similarly, symptomatic infants of Scimitar syndrome in our center presented with more chance of developing PH, PVS, and mortality, suggesting that symptomatic Scimitar syndrome infants are appropriate surgical candidates and should undergo surgery at an early stage. Nevertheless, the optimal timing for surgical repair in infants remains poorly defined and demands further study (10).

Corrective surgical procedures include baffling a tunnel or direct re-implantation of the aberrant pulmonary venous drainage into the LA. For many years, both baffle repair and direct reimplantation of the Scimitar vein have been described. They are chosen mainly depending on the surgeon's preference and each patient's anatomic and pathological aspects. There is still no consensus on the optimal surgical treatment approach (22–24). In the present study, we found that corrective surgery could be done safely with a low mortality and morbidity rate. There was no significant difference in age at surgery, cardiopulmonary bypass time, ICU staying time, and postoperative complications between these two surgical techniques. A European Congenital Heart Surgeons Association (ECHSA) multicentric study revealed that although the direct reimplantation technique is linked to a higher incidence of postoperative complications and a longer hospital stay, the overall freedom from Scimitar vein stenosis was 84.5% at 10 years and was similar between patients who had intracardiac baffle repair and reimplantation of the Scimitar vein (83.8% vs. 85.8%) (10).

PVS is considered one of the most common complications of perioperative Scimitar syndrome patients, regardless of the redirection technique. It's reported that 25% of Scimitar vein stenosis or Scimitar vein drainage occlusion has been observed in the postoperative period, mostly in newborns (25). Due to persistent restenosis, percutaneous and surgical treatments of PVS have poor success, and mortality remains high (26). It has been reported that compared with those with direct anastomosis, infantile form of Scimitar syndrome patients who received intra-atrial baffle access are more tended to occur Scimitar vein obstruction, most of whom require reoperation (12). Inconsistent with this finding, our data demonstrated that there was no significant difference in the PVS incidence between baffle repair and Scimitar vein reimplantation methods, and the occurrence of post-operation PVS was related to pulmonary vessel development before operation instead. However, the long-term outcomes of different operation options and post-operative PVS treated with balloon dilation or stent implantation still need further evaluation.

Symptomatic patients under 1 year are typically sick, with a high surgical mortality and complication rate. In contrast, older people have better immediate and long-term results (10). ECHSA multicenter study revealed that the patient's age influences the outcome of surgical treatment of Scimitar syndrome (10). A higher mortality rate is linked to the existence of PH. The fact that direct reimplantation is connected to a higher incidence of postoperative complications and a longer hospital stay is also noteworthy. Consistently, our data indicate that the mortality of Scimitar syndrome is linked to age at diagnosis, PH, and PVS. All the death were within 4 months old, complicated with PVS, tracheal stenosis, APCs, left to right shunting congenital heart disease, and other factors leading to PH.

However, our study still has limitations since this is a retrospective analysis with varieties and biases of patients' characteristics, therapeutic techniques used, and period of the populations. Pulmonary scintigraphy is not performed in all patients, especially for postoperative right lung perfusion assessment. We believe radiological examination during long-term follow-up should be performed routinely to achieve a more accurate evaluation. Besides, the population enrolled in current study is still not large enough, further study concerning personalized management with larger population of Scimitar syndrome on a prospective basis is required.

To summarize, Scimitar syndrome is a rare congenital anomaly that needs multidisciplinary and sequential assessment for early referral. Interventional treatment of Scimitar syndrome candidates an effective therapy to alleviate pulmonary artery pressure and progression during infancy. Baffle repair and direct reimplantation of the Scimitar vein used in the surgical treatment of Scimitar syndrome are safe and have similar effects. Age at diagnosis, PH, and PVS are high-risk factors for death in Scimitar syndrome.

## Data availability statement

The original contributions presented in the study are included in the article/supplementary material, further inquiries can be directed to the corresponding author.

## Ethics statement

The studies involving human participants were reviewed and approved by Ethics Committee of Shanghai Children's Medical Center. Written informed consent to participate in this study was provided by the participants' legal guardian/next of kin. Written informed consent was obtained from the individual(s), and minor(s)' legal guardian/next of kin, for the publication of any potentially identifiable images or data included in this article.

## Author contributions

KW, XX, and YG conceived and designed the study. KW and XX collected clinical data. KW, XX, TL, and WG analyzed the data. KW and YG wrote the manuscript. XX, TL, and WG edited the manuscript with important intellectual content. YG supervised this study. All authors contributed to the article and approved the submitted version.

## Funding

This study was supported by the Natural Science Foundation of Zhejiang Province (LY22H070005) and a Grant from the Science and Technology Bureau of Wenzhou (Y20210010).

## Conflict of interest

The authors declare that the research was conducted in the absence of any commercial or financial relationships that could be construed as a potential conflict of interest.

## Publisher's note

All claims expressed in this article are solely those of the authors and do not necessarily represent those of their affiliated organizations, or those of the publisher, the editors and the reviewers. Any product that may be evaluated in this article, or claim that may be made by its manufacturer, is not guaranteed or endorsed by the publisher.
